# Amivantamab plus lazertinib in osimertinib-relapsed *EGFR*-mutant advanced non-small cell lung cancer: a phase 1 trial

**DOI:** 10.1038/s41591-023-02554-7

**Published:** 2023-09-14

**Authors:** Byoung Chul Cho, Dong-Wan Kim, Alexander I. Spira, Jorge E. Gomez, Eric B. Haura, Sang-We Kim, Rachel E. Sanborn, Eun Kyung Cho, Ki Hyeong Lee, Anna Minchom, Jong-Seok Lee, Ji-Youn Han, Misako Nagasaka, Joshua K. Sabari, Sai-Hong Ignatius Ou, Patricia Lorenzini, Joshua M. Bauml, Joshua C. Curtin, Amy Roshak, Grace Gao, John Xie, Meena Thayu, Roland E. Knoblauch, Keunchil Park

**Affiliations:** 1 Division of Medical Oncology, Yonsei Cancer Center, Yonsei University College of Medicine, Seoul, Republic of Korea; 2 Seoul National University College of Medicine and Seoul National University Hospital, Seoul, Republic of Korea; 3 Virginia Cancer Specialists Research Institute, US Oncology Research, Fairfax, VA USA; 4 Icahn School of Medicine at Mount Sinai, New York, NY USA; 5 H. Lee Moffitt Cancer Center & Research Institute, Tampa, FL USA; 6 Asan Medical Center, University of Ulsan College of Medicine, Seoul, Republic of Korea; 7 Earle A. Chiles Research Institute, Providence Cancer Institute, Portland, OR USA; 8 Gil Medical Center, Gachon University College of Medicine, Incheon, Republic of Korea; 9 Chungbuk National University Hospital, Cheongju, Republic of Korea; 10 Drug Development Unit, Royal Marsden/Institute of Cancer Research, Sutton, UK; 11 Seoul National University Bundang Hospital, Seongnam, Republic of Korea; 12 National Cancer Center, Gyeonggi-do, Republic of Korea; 13 University of California Irvine School of Medicine, Chao Family Comprehensive Cancer Center, Orange, CA USA; 14 New York University School of Medicine, New York, NY USA; 15 Janssen R&D, Spring House, PA USA; 16 Samsung Medical Center, Sungkyunkwan University School of Medicine, Seoul, Republic of Korea; 17 Present Address: Department of Thoracic/Head and Neck Medical Oncology, The University of Texas MD Anderson Cancer Center, Houston, TX USA

**Keywords:** Predictive markers, Clinical trials, Small-cell lung cancer

## Abstract

Patients with epidermal growth factor receptor (*EGFR*)-mutated non-small cell lung cancer (NSCLC) often develop resistance to current standard third-generation EGFR tyrosine kinase inhibitors (TKIs); no targeted treatments are approved in the osimertinib-relapsed setting. In this open-label, dose-escalation and dose-expansion phase 1 trial, the potential for improved anti-tumor activity by combining amivantamab, an EGFR-MET bispecific antibody, with lazertinib, a third-generation EGFR TKI, was evaluated in patients with *EGFR*-mutant NSCLC whose disease progressed on third-generation TKI monotherapy but were chemotherapy naive (CHRYSALIS cohort E). In the dose-escalation phase, the recommended phase 2 combination dose was established; in the dose-expansion phase, the primary endpoints were safety and overall response rate, and key secondary endpoints included progression-free survival and overall survival. The safety profile of amivantamab and lazertinib was generally consistent with previous experience of each agent alone, with 4% experiencing grade ≥3 events; no new safety signals were identified. In an exploratory cohort of 45 patients who were enrolled without biomarker selection, the primary endpoint of investigator-assessed overall response rate was 36% (95% confidence interval, 22–51). The median duration of response was 9.6 months, and the median progression-free survival was 4.9 months. Next-generation sequencing and immunohistochemistry analyses identified high EGFR and/or MET expression as potential predictive biomarkers of response, which will need to be validated with prospective assessment. ClinicalTrials.gov identifier: NCT02609776.

## Abstract (web summary)

The combination of a bispecific antibody against EGFR and MET with a tyrosine kinase inhibitor (TKI) in patients with TKI-relapsed, chemotherapy-naive, EGFR-mutated advanced NSCLC is safe and shows preliminary efficacy.

## Main

Mutations in the epidermal growth factor receptor (*EGFR*) are among the most common activating mutations in non-small cell lung cancer (NSCLC), with exon 19 deletions (ex19del) and exon 21 L858R mutations accounting for approximately 85–90% of all cases^1,2^. The introduction of EGFR tyrosine kinase inhibitors (TKIs) to treat *EGFR*-mutant NSCLC has led to marked improvements in clinical outcomes, with response rates of 60–80%^3–9^. Osimertinib, a third-generation EGFR TKI, is the current standard of care for the treatment of *EGFR* ex19del and L858R NSCLC, with demonstrated median progression-free survival (PFS) and overall survival (OS) of 18.9 months and 38.6 months, respectively^9,10^. Despite good initial disease control, patients nearly always develop resistance to osimertinib. Recent studies have evaluated chemotherapy plus immunotherapy and anti-angiogenic therapy in this patient population^11^, but no subsequent targeted therapeutic approaches without chemotherapy are approved in the osimertinib-relapsed setting.

Based on next-generation sequencing (NGS) of circulating tumor DNA (ctDNA) and tumor samples from patients who experience disease progression on osimertinib, identified mechanisms of resistance can be broadly divided into EGFR-dependent mechanisms (alterations preventing osimertinib inhibition of EGFR) and EGFR-independent mechanisms (activation of alternate signaling pathways or reprogramming, such as epithelial–mesenchymal transition and histologic transformations)^12,13^. The most prevalent EGFR-dependent mechanism of resistance to osimertinib is C797S mutation of the *EGFR* gene, which abrogates binding of osimertinib to the ATP binding site in the kinase domain^14–16^. Other EGFR-dependent resistance mechanisms that have been identified include L792X, G796X, L718Q and *EGFR* amplification^12,13,15,17–19^. Among EGFR-independent resistance mechanisms, *MET* amplification has been most frequently reported, with activation of mitogen-activated protein kinase or phosphatidylinositol 3-kinase pathways, gene fusions and histologic transformations also reported^15,17–19^. However, in up to 50% of patients who experience progression on osimertinib, no clear mechanism of resistance has been identified^12,13^.

Overcoming osimertinib resistance is further complicated by heterogeneous patterns of resistance and presence of co-occurring resistance mechanisms, which can occur even within a single patient^20^. Additionally, the mechanism of osimertinib resistance can be influenced by whether progression occurred in the first-line or second-line (post-EGFR TKI, T790M^+^) setting^8,15,17–19^. Given the complexity of osimertinib patterns of resistance, the inherent resistance of this population to immuno-oncology (IO) monotherapy and the lack of approved targeted therapies, current treatment guidelines recommend platinum-based chemotherapy regimens after progression on osimertinib^21–23^.

Amivantamab is a fully human bispecific antibody that binds to the EGFR and MET receptor to inhibit ligand binding, promote downregulation of cell surface receptors and induce Fc-dependent trogocytosis and antibody-dependent cellular cytotoxicity^24–27^. Amivantamab has shown anti-tumor activity across diverse EGFR-driven and MET-driven NSCLC^28,29^, with a tolerable safety profile, and is approved for the treatment of patients with locally advanced or metastatic NSCLC with *EGFR* exon 20 insertion mutations, whose disease progressed on or after platinum-based chemotherapy^28,30–32^. Amivantamab, by binding extracellularly, provides a complementary mechanism to EGFR TKIs, with the combination simultaneously targeting both the extracellular and intracellular catalytic domains of EGFR. This potential for improved patient outcomes has been demonstrated in preclinical studies in the murine H1975-HGF xenograft model where greater tumor reductions and more durable disease control were observed when amivantamab was given in combination with lazertinib, a potent brain-penetrant third-generation EGFR TKI with efficacy against activating *EGFR* and T790M mutations, as compared to treatment with either agent alone^33^. Given the tolerable safety profiles of both amivantamab and lazertinib and the potential for improved anti-tumor activity, the amivantamab and lazertinib regimen was evaluated in the ongoing CHRYSALIS study, with preliminary efficacy assessed in patients with *EGFR* ex19del or L858R metastatic NSCLC whose disease progressed on osimertinib or another third-generation EGFR TKI but had not received cytotoxic chemotherapy in the metastatic setting (cohort E).

## Results

### Patients

**Fig. 1:**
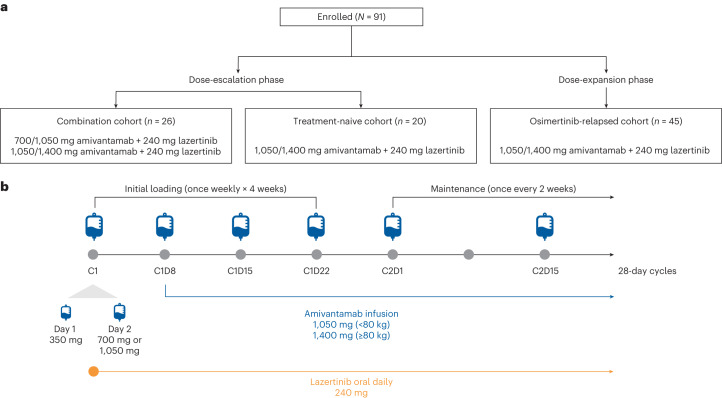
Patient flow diagram and regimen dosing schema. **a**, Patient flow for the three cohorts from the dose-escalation and dose-expansion phases of CHRYSALIS. **b**, Dosing schema for amivantamab and lazertinib. Blue symbols indicate intravenous administration of an amivantamab dose.

As of the data cutoff date of 19 April 2021 (enrollment start date, 3 December 2019), a total of 91 patients across three different cohorts from both the dose-escalation and dose-expansion phases of the CHRYSALIS study have received the amivantamab and lazertinib regimen (Fig. 1). In the dose-escalation phase, the combination cohort (*n* = 26), which was investigated only at sites in Korea, enrolled patients without restriction on prior therapies to evaluate amivantamab at an initial dose of 700 mg (1,050 mg for body weight ≥80 kg), followed by a second dose level of 1,050 mg (1,400 mg for body weight ≥80 kg) in combination with 240 mg of lazertinib. No dose-limiting toxicity was observed in the dose-escalation phase, and the recommended phase 2 combination dose (RP2CD) of 1,050 mg (1,400 mg for body weight ≥80 kg) of amivantamab + 240 mg of lazertinib was selected. After determination of the RP2CD, the Safety Evaluation Team agreed to further assess the tolerability of the RP2CD in a second cohort in Korea, which enrolled treatment-naive patients (*n* = 20). In parallel, the osimertinib-relapsed cohort (also known as cohort E) in the dose-expansion phase of the study enrolled patients globally whose disease had relapsed on osimertinib without intervening platinum-based chemotherapy (*n* = 45; Fig. 1a). The analysis presented here will focus on this osimertinib-relapsed cohort; however, the safety analysis will also include all 91 patients who received the amivantamab and lazertinib regimen in CHRYSALIS (combination cohort (*n* = 26), treatment-naive patients (*n* = 20) and the osimertinib-relapsed cohort (*n* = 45, also known as cohort E)). A full analysis of the other populations will be published separately.

The baseline patient demographics and disease characteristics for the osimertinib-relapsed cohort and all-treated population are presented in Table 1 (see Supplementary Table 1 for demographics and baseline disease characteristics for the combination cohort). In the osimertinib-relapsed cohort, the median age was 65 years (minimum–maximum, 39–85); 25 patients (56%) were women; and 19 patients (42%) were Asian. More patients harbored ex19del (69%) than L858R (31%) intrinsic mutations. Patients received a median number of two prior lines of therapy; all patients received a third-generation EGFR TKI, which was received as second-line therapy in 73% of patients. Thirteen patients (29%) had a history of brain lesions before receiving the first study dose.

**Table 1:** Demographic and baseline disease characteristics

	Osimertinib-relapsed (*n* = 45)	All-treated population (*N* = 91)
Median age, years (minimum–maximum)	65 (39–85)	61 (36–85)
Sex		
Female	25 (56)	52 (57)
Male	20 (44)	39 (43)
Race		
Asian	19 (42)	65 (71)
White	20 (44)	20 (22)
Black	2 (4)	2 (2)
Multiple/not reported	4 (9)	4 (4)
ECOG PS		
0	12 (27)	29 (32)
1	33 (73)	62 (68)
History of smoking		
Yes	20 (44)	41 (45)
No	25 (56)	50 (55)
Median time from initial diagnosis to first dose, months (minimum–maximum)	32 (5–98)	24 (1–98)
Location of metastases^a^		
Lymph node	18 (40)	44 (48)
Bone	19 (42)	31 (34)
Brain	13 (29)	30 (33)
Liver	8 (18)	10 (11)
Adrenal gland	4 (9)	4 (4)
Other/not reported	22 (49)	47 (52)
Median prior lines of therapy (minimum–maximum)	2 (1–4)	2 (0–9)
*EGFR* primary mutation		
Exon 19 deletion	30 (67)	—
Exon 21 L858R	14 (31)	—
Unknown	1 (2)	—
Prior systemic therapy	45 (100)	—
Platinum-based chemotherapy^b^	7 (16)	18 (20)
EGFR TKI^a^		
1st or 2nd generation	33 (73)	54 (59)
3rd generation	45 (100)	53 (58)
Received as 1st line	12 (27)	—
Received as 2nd line	33 (73)	—
No prior therapy	0	23 (25)

Data are number of patients (%) unless otherwise noted.

^a^Patients could be counted in more than one category.

^b^Seven patients had limited platinum exposure (<two cycles) given before first EGFR TKI in the osimertinib-relapsed group.

### Safety

At the 19 April 2021 data cutoff, the median duration of follow-up was 11.1 months (minimum–maximum, 1.0–15.0) for the osimertinib-relapsed cohort and 13.3 months (minimum–maximum, 0.5–23.7) for the all-treated population. The safety profile of the amivantamab and lazertinib regimen was similar in both of these cohorts and generally similar to safety previously described for amivantamab at its recommended phase 2 dose (RP2D) (ref. ^31^). Adverse events (AEs) reported in the dose-escalation combination cohort are presented in Supplementary Table 2.

In the osimertinib-relapsed cohort, rash-related AEs occurred in 36 patients (80%), with two patients (4%) experiencing grade ≥3 events (Table 2). Infusion-related reaction (IRR) was reported in 35 patients (78%) who all had events of grade 1 or 2 severity. IRRs occurred with the initial infusion on cycle 1, day 1 and did not lead to treatment discontinuations. Other frequently reported AEs were consistent with on-target anti-EGFR and anti-MET activity. AEs traditionally associated with EGFR inhibition included paronychia in 22 patients (49%), pruritus in 14 patients (31%), stomatitis in 12 patients (27%) and diarrhea in 10 patients (22%) (Table 2). AEs traditionally associated with MET inhibition of hypoalbuminemia and edema occurred in 17 patients (38%) each (Table 2). AEs of grade ≥3 severity were reported in 25 patients (56%), with seven patients (16%) experiencing grade ≥3 AEs that were considered to be treatment related (related to either or both amivantamab and lazertinib). The most common treatment-related grade ≥3 AEs were increased alanine aminotransferase (ALT) and paronychia, both reported in two patients (4%) each; both increased ALT events were resolved without treatment discontinuation. Serious AEs occurred in 17 patients (38%), of whom two (4%; one pneumonitis and one dermatitis) had events that were considered to be treatment related. Treatment-related AEs that led to dose reduction and treatment discontinuation of any study agent occurred in eight patients (18%; one increased ALT, one increased aspartate aminotransferase (AST), one headache, three paronychia, two rash and three dermatitis acneiform) and two patients (4%; one pneumonitis and one dermatitis), respectively. Treatment-related dose interruptions of any study agent occurred in 12 patients (27%). In one patient with worsening dyspnea, an unscheduled computed tomography scan at 4 weeks documented grade 3 pneumonitis in the setting of rapidly progressive disease (PD) in the left lung. Given the disease burden, the patient was not a candidate for intubation and died shortly after presentation, with death attributed to both PD and pneumonitis. Overall, no increased risk of pneumonitis or new safety signals were identified.

**Table 2:** Adverse events

Adverse events (≥10%), *n* (%)	Osimertinib-relapsed (*n* = 45)		All-treated (*N* = 91)	
	All-grade	Grade ≥3	All-grade	Grade ≥3
Skin and subcutaneous tissue disorders				
Rash^a^	36 (80)	2 (4)	81 (89)	6 (7)
Pruritus	14 (31)	0	31 (34)	0
Dry skin	13 (29)	0	16 (18)	0
Skin fissures	7 (16)	0	8 (9)	0
General disorders and administration-site conditions				
Infusion-related reaction	35 (78)	0	60 (66)	1 (1)
Edema^b^	17 (38)	0	25 (27)	0
Fatigue^c^	12 (27)	0	21 (23)	1 (1)
Pyrexia	6 (13)	0	12 (13)	0
Infections and infestations				
Paronychia	22 (49)	2 (4)	58 (64)	4 (4)
Metabolism and nutrition disorders				
Hypoalbuminemia	17 (38)	1 (2)	42 (46)	4 (4)
Decreased appetite	6 (13)	0	19 (21)	0
Hypocalcemia	9 (20)	0	14 (15)	1 (1)
Hypomagnesemia	6 (13)	0	9 (10)	0
Hyponatremia	5 (11)	1 (2)	8 (9)	4 (4)
Musculoskeletal and connective tissue disorders				
Musculoskeletal pain^d^	19 (42)	1 (2)	39 (43)	1 (1)
Muscle spasms	5 (11)	0	8 (9)	0
Gastrointestinal disorders				
Stomatitis^e^	12 (27)	0	34 (37)	0
Nausea	20 (44)	0	28 (31)	1 (1)
Constipation	12 (27)	0	19 (21)	0
Diarrhea	10 (22)	0	17 (19)	1 (1)
Dyspepsia	3 (7)	0	12 (13)	0
Vomiting	9 (20)	0	10 (11)	0
Investigations				
Increased ALT	8 (18)	2 (4)	29 (32)	5 (5)
Increased AST	10 (22)	0	26 (29)	2 (2)
Increased blood alkaline phosphatase	5 (11)	0	6 (7)	0
Nervous system disorders				
Paresthesia	5 (11)	0	23 (25)	0
Dizziness	10 (22)	0	19 (21)	0
Headache^f^	9 (20)	1 (2)	11 (12)	1 (1)
Respiratory, thoracic and mediastinal disorders				
Dyspnea^g^	11 (24)	3 (7)	15 (16)	4 (4)
Pulmonary embolism	4 (9)	3 (7)	11 (12)	3 (3)
Cough^h^	4 (9)	0	10 (11)	0
Vascular disorders				
Hemorrhage^i^	6 (13)	0	10 (11)	0
Hypotension	5 (11)	0	6 (7)	0
Blood and lymphatic system disorders				
Thrombocytopenia	6 (13)	0	8 (9)	0
Psychiatric disorders				
Anxiety	5 (11)	0	5 (5)	0

^a^Rash includes acne, dermatitis, dermatitis acneiform, eczema, eczema asteatotic, palmar–plantar erythrodysesthesia syndrome, perineal rash, rash, rash erythematous, rash maculo-papular, rash papular, rash vesicular, skin exfoliation and toxic epidermal necrolysis.

^b^Edema includes eyelid edema, face edema, generalized edema, lip edema, edema, edema peripheral, periorbital edema and peripheral swelling.

^c^Fatigue includes asthenia and fatigue.

^d^Musculoskeletal pain includes arthralgia, arthritis, back pain, bone pain, musculoskeletal chest pain, musculoskeletal discomfort, musculoskeletal pain, myalgia, neck pain, non-cardiac chest pain, pain in extremity and spinal pain.

^e^Stomatitis includes aphthous ulcer, cheilitis, glossitis, mouth ulceration, mucosal inflammation, pharyngeal inflammation and stomatitis.

^f^Headache includes headache and migraine.

^g^Dyspnea includes dypsnea and dyspnea exertional.

^h^Cough includes cough, productive cough and upper airway cough syndrome.

^i^Hemorrhage includes epistaxis, gingival bleeding, hematuria, hemoptysis, hemorrhage, mouth hemorrhage and mucosal hemorrhage.

### Efficacy

**Fig. 2:**
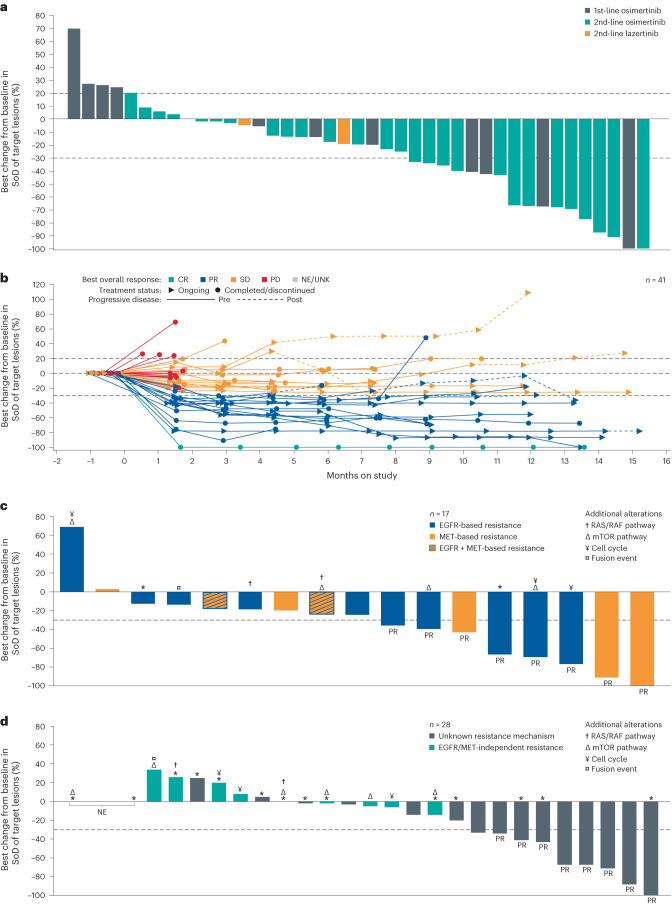
Anti-tumor activity of amivantamab + lazertinib combination in part 2 expansion cohort E: osimertinib-relapsed NSCLC with common *EGFR* mutations (panels a and b) and among patients with and without identified EGFR-based and/or MET-based resistance (panels c and d). **a**, Waterfall plot displaying best percent change from baseline in sum of lesion diameters among patients enrolled in the osimertinib-relapsed cohort by receipt of osimertinib/lazertinib as first-line (yellow) or second-line (blue/green) therapy. Teal bars denote patients who received the third-generation EGFR TKI lazertinib instead of osimertinib. Four patients did not have any post-baseline disease assessments and are not included in the plot. **b**, Spider plot displaying percent change from baseline in sum of diameters of target lesions over time in patients enrolled in the osimertinib-relapsed cohort. Best response of CR (green), PR (blue), SD (orange) and PD (red) are indicated. Gray lines represent patients who were not evaluable (NE). Four patients did not have any post-baseline disease assessments and are not included in the plot. **c**, Waterfall plot displaying best percent change from baseline in sum of diameters of target lesions among 17 patients with identified EGFR-based and MET-based osimertinib resistance mechanisms. **d**, Waterfall plot displaying best percent change from baseline in sum of diameters of target lesions among 28 patients with unknown or EGFR-independent and MET-independent osimertinib resistance mechanisms identified by NGS. Additional alterations identified in each patient are indicated by the symbols. Asterisks denote patients who did not have tumor NGS. SoD, sum of diameters; UNK, unknown.

**Extended Data Fig. 1:**
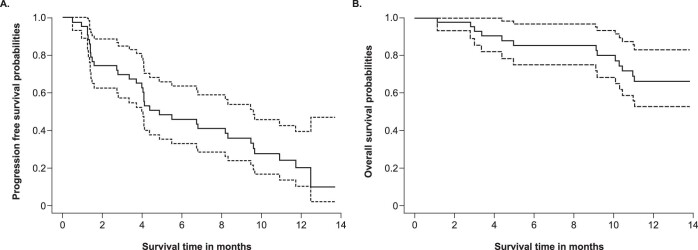
(A) PFS and (B) OS K-M Curve. In both K-M curves, the dotted lines represent the 95% confidence intervals. K-M, Kaplan-Meier; OS, overall survival; PFS, progression free survival.

At a median follow-up of 11.1 months, the investigator-assessed overall response rate (ORR) in the osimertinib-relapsed cohort was 36% (95% confidence interval (CI), 22–51) with one complete response (CR) and 15 partial responses (PRs) (Table 3). ORRs were similar between patients who had received osimertinib as either first-line or second-line therapy (ORR of 33% (95% CI, 10–65) and 36% (95% CI, 21–55), respectively; Fig. 2a). For patients with *EGFR* ex19 del (*n* = 30) or L858R (*n* = 14), the ORR was 33% (95% CI, 17–53) and 43% (95% CI, 18–71), respectively. Most responses (14/16) were observed at the first disease assessment at 6 weeks. The median duration of response was 9.6 months (95% CI, 5.3–not calculable (NC)), with 11 patients (69%) achieving responses lasting ≥6 months (Fig. 2b). The clinical benefit rate (CBR), defined as CR, PR or stable disease (SD) for ≥11 weeks, was 64% (95% CI, 49–78). The median PFS was 4.9 months (95% CI, 3.7–9.5); for patients who had received osimertinib as either first-line or second-line therapy, median PFS was 6.8 months and 2.9 months, respectively (Extended Data Fig. 1a). Median OS was NC (Extended Data Fig. 1b). In total, three patients had documented central nervous system (CNS) progression, two with new lesions and one with progression of an existing lesion.

**Table 3:** Investigator-assessed response per RECIST

	Osimertinib-relapsed (*n* = 45)
ORR^a^ (95% CI)	36% (22–51)
CBR^b^ (95% CI)	64% (49–78)
Best response, *n* (%)	
CR	1 (2)
PR	15 (33)
SD	14 (31)
PD	11 (24)
NE	4 (9)
mDOR, months (95% CI)	9.6 (5.3–NC)
mPFS, months (95% CI)	4.9 (3.7–9.5)
mOS, months (95% CI)	NC

^a^Proportion of patients who had CRs or PRs.

^b^Proportion of patients who had CRs or PRs or SD for ≥11 weeks (corresponding to two disease assessments).

mDOR, median duration of response; mOS, median overall survival; mPFS, median progression-free survival; NE, not evaluable.

### Biomarker analyses

Given the known heterogeneity of osimertinib resistance, NGS was used to better understand tumor response to the amivantamab and lazertinib regimen and to explore potential biomarkers predictive of response in the osimertinib-relapsed cohort. Patient ctDNA and tumor tissue were available for NGS analysis in 44 of 45 patients and 29 of 45 patients, respectively. Genetic testing of these samples identified 17 patients (38%) who had EGFR-based and/or MET-based osimertinib resistance mutations or amplifications (Fig. 2c and Supplementary Table 3). Associated biomarker data for all 45 patients are provided in Supplementary Table 4. The most frequent alterations identified were *EGFR* C797S (*n* = 7; all *cis*); *MET* amplification (*n* = 5), with copy number variation (CNV) of 3, 4 (*n* = 2), 7 and 31; *EGFR* amplification (*n* = 3), with CNV of 8, 14 and 37; and *EGFR* L718X (*n* = 3) (Supplementary Table 3). Seven of these patients harbored more complex, heterogeneous alterations comprising both EGFR- and/or MET-dependent and -independent resistance mechanisms, including alterations in PIK3CA, KRAS and components of the cell cycle machinery. One patient harbored an FGFR3–TACC3 fusion in addition to an *EGFR* C797S mutation.

Among the 17 patients with EGFR-based and/or MET-based osimertinib resistance, eight achieved a response based on investigator assessment for an ORR of 47% (95% CI, 23–72), with a median duration of response of 10.4 months (95% CI, 2.7–NC). The CBR was 82% (95% CI, 57–96), and the median PFS was 6.7 months (95% CI, 3.4–12.5) (Supplementary Table 5). Three of five patients (60%) who were observed to have *MET* amplification after progression on osimertinib had confirmed responses to the amivantamab and lazertinib regimen, including one patient with a CR (Supplementary Table 6). Different response patterns were observed depending on the co-occurring EGFR/MET-independent resistance mechanisms, with responses observed in two of three patients with concurrent PIK3CA alterations and two of three patients with concurrent alterations in cell cycle machinery. Responses were not observed in patients with concurrent KRAS alterations or in the patient with an FGFR–TACC3 fusion (Supplementary Table 6).

Of the remaining 28 patients who did not have an identified EGFR-based and/or MET-based osimertinib resistance mechanism, 18 had unknown mechanisms (of these, one had neither tissue nor ctDNA and 13 had ctDNA testing but no tumor testing), and 10 had EGFR-independent and/or MET-independent resistance mechanisms, such as alterations in PIK3CA, KRAS and PTEN, and mutations in cell cycle genes, identified by NGS (Fig. 2d and Supplementary Table 7). The investigator-assessed ORR in this subgroup of patients was 29% (95% CI, 13–49), with eight of 28 patients achieving responses. The median duration of response was 8.3 months (95% CI, 2.6–NC). The CBR was 54% (95% CI, 34–73), and the median PFS was 4.1 months (95% CI, 1.4–9.5) (Supplementary Table 5). Among the 18 patients with unknown mechanisms of resistance, the ORR was 44% (95% CI, 22–69), and, among the 10 patients with EGFR-independent and/or MET-independent resistance mechanisms, no patient achieved a response. Of note, all eight patients who had a PR had unknown mechanisms of osimertinib resistance by NGS.

**Fig. 3:**
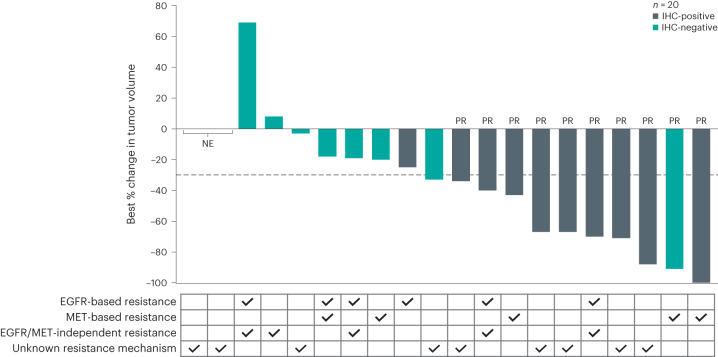
Anti-tumor activity in patients by IHC expression analysis and NGS-identified osimertinib resistance mechanisms. Waterfall plot displaying best percent change from baseline in sum of diameters of target lesions among 20 patients who had tumor samples available for exploratory analysis using IHC staining for EGFR and MET expression. IHC-positive patients had combined EGFR and MET H-scores ≥400, and IHC-negative patients had combined EGFR and MET H-scores <400. The table below the waterfall plot indicates the type of resistance mechanism identified using NGS. Patients with both EGFR-based and EGFR/MET-independent resistance are categorized as having EGFR-based resistance (Fig. 2a).

**Extended Data Fig. 2:**
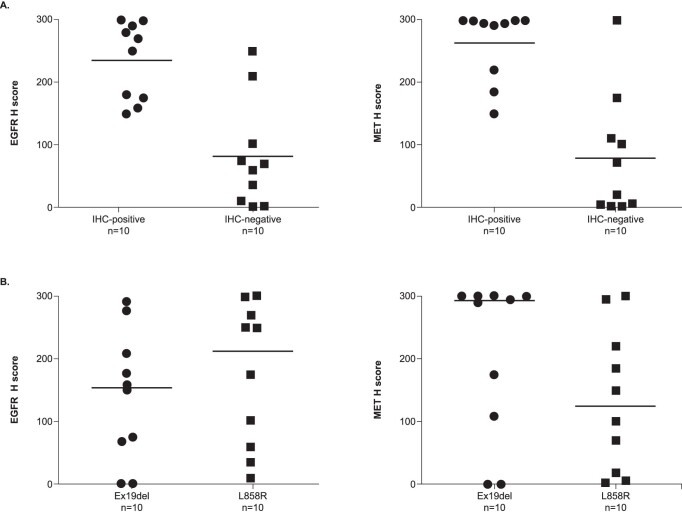
Distribution of EGFR and MET H-Scores by (A) IHC status or (B) *EGFR* mutation type. In each set of data points, the middle bar represents the mean. *EGFR*, epidermal growth factor receptor; Ex19del, Exon 19 deletion; IHC, immunohistochemistry.

In addition to NGS, an immunohistochemistry (IHC)-based approach was undertaken in patients with sufficient remaining tumor samples (*n* = 20) to explore the association of EGFR and MET expression with tumor response. Representative images of IHC staining are provided in the supplement. By IHC testing, 10 patients were identified as having a combined H-score ≥400, up to a maximum 600 (referred to hereafter as ‘IHC-positive’) for EGFR and/or MET expression (Fig. 3). All patients who were IHC-positive had an H-score ≥150, of a maximum of 300, for both EGFR and MET. The average EGFR H-score in IHC-positive patients was 235, whereas the average in IHC-negative patients was 82. Similarly, average MET H-score in IHC-positive patients was 264 but only 78 in IHC-negative patients; breakdown by mutation type is also provided (Extended Data Fig. 2). Of the 20 patients included in this analysis, 10 had a confirmed PR (Fig. 3). In the 10 patients who were IHC-positive, nine had PRs, for an ORR of 90% (95% CI, 56–100) and a median duration of response of 9.7 months (95% CI, 2.6–NC). The CBR was 100% (95% CI, 69–100), and the median PFS was 12.5 months (95% CI, 4.0–NC) (Supplementary Table 5). Among the 10 patients who were IHC-negative, only one achieved a PR, for an ORR of 10% (95% CI, 0.3–45) and a duration of response of 2.7 months (95% CI, NC). The CBR was 50% (95% CI, 19–81), and the median PFS was 4.0 months (95% CI, 1.4–4.4). Although NGS has its utility, the IHC-positive cohort seemed to additionally identify a disparate patient population from NGS testing—responders who were IHC-positive included patients with genetic EGFR- and/or MET-dependent and -independent resistance as well as those with unknown resistance mechanism by NGS (Fig. 3).

## Discussion

Patients with *EGFR* ex19del and L858R mutations receive osimertinib as part of standard-of-care therapy, in either the first-line or second-line setting; upon progression after osimertinib, the standard of care is platinum-based chemotherapy. Furthermore, salvage therapy with docetaxel after chemotherapy offers an ORR of only 14% (ref. ^34^), highlighting the need for additional therapies that can prolong disease control. Amivantamab’s mode of action, initiated through binding to the extracellular domain of EGFR and MET receptors, has the potential to target both EGFR-dependent and MET-dependent mechanisms of osimertinib resistance and, also in concert with tyrosine kinase inhibition by lazertinib, may lead to more potent inhibition of EGFR oncogenic signaling. The combination of TKIs targeting EGFR (osimertinib) and MET (savolitinib or tepotinib) in this patient population has similarly shown the benefit of targeting these pathways in *EGFR*-mutant NSCLC^35,36^.

Overall, the safety profile of the amivantamab and lazertinib regimen was tolerable and generally consistent with previous monotherapy experience of amivantamab^31,37^, demonstrating that the favorable safety profile of lazertinib enables combination with amivantamab. Among all patients treated with the amivantamab and lazertinib regimen, the most commonly reported toxicities were rash (89%) and IRRs (66%); the incidence of rash was higher than that previously reported with amivantamab (78%) or lazertinib monotherapy (37%) (refs. ^31,38^). Most on-target toxicities were of grade 1 or 2 severity, with low rates of grade ≥3 rash (7%) and diarrhea (1%) reported. There was no evidence of increased risk of pneumonitis, and no new safety signals were identified.

In the osimertinib-relapsed cohort, which was enrolled without biomarker selection, the investigator-assessed ORR was 36%, with anti-tumor activity observed in patients whose disease progressed after osimertinib therapy in the first-line or second-line setting. Furthermore, the median PFS was 4.9 months, which is similar to that observed with standard-of-care platinum-based chemotherapy^39^. In this population, the ORR for those with *EGFR* ex19 del (33%) and L858R (43%) was roughly numerically equivalent. Exploratory analysis by NGS identified EGFR-based and MET-based resistance mechanisms as potential biomarkers for response. However, half of the responders had unknown mechanisms of resistance or lower sensitivity in ctDNA (there were eight unknown responders; five of eight had both ctDNA and tumor NGS performed, and three of eight had ctDNA only), suggesting that reliance on NGS alone could potentially miss patients who might benefit from the amivantamab and lazertinib regimen. An exploratory IHC-based approach showed a potential association between high EGFR and/or MET expression and response to the amivantamab and lazertinib regimen. Retrospective IHC-based analysis appeared to have a stronger correlation with response than NGS, identifying responders who had EGFR- and/or MET-dependent and -independent resistance mechanisms as well as those who had mechanisms that were unknown. Notably, five of the nine responders did not have a clear genetic resistance mechanism, suggesting that IHC testing may identify potential responders despite the absence of an identifiable genetic resistance mechanism. Among the 16 responders in the osimertinib-relapsed cohort, eight had EGFR-based and/or MET-based resistance, and eight did not have resistance mechanisms identified through NGS, suggesting that at least some of the tumors with unknown resistance may reflect non-genetic mechanisms leading to TKI resistance but continued sensitivity to EGFR-directed and MET-directed inhibition by the combined action of amivantamab and lazertinib or the immune-based anti-tumor effects of amivantamab. Although promising, it should be noted that the H-score cutoffs for the determination of IHC-positive patients were determined retrospectively, and these potential biomarker strategies are being prospectively explored in the ongoing phase 1/1b CHRYSALIS-2 study (NCT04077463).

This study needs to be interpreted within its limitations. As a non-randomized, single-arm trial with no control arm, interpretation of the data requires historical comparison within the literature or with real-world evidence. The limited sample size of the study leads to lower-than-desired precision, which can impact interpretation and extrapolation. Additionally, the data presented here are not generalizable to all patients who progressed on osimertinib because the study enrolled only those who were also chemotherapy naive. Long-term safety of the amivantamab and lazertinib combination therapy may not be fully captured with the follow-up period explored in this study. Data with longer follow-up (median follow-up of 33.5 months) from this same study and combination in the front-line setting were recently presented, and the safety profile was consistent with previous reports^40^. Additionally, the ongoing phase 3 trials (MARIPOSA (NCT04487080), MARIPOSA-2 (NCT04988295) and PALOMA-3 (NCT05388669)) will provide a more comprehensive long-term representation of the safety of amivantamab and lazertinib combination therapy.

The activity of the combination after disease progression on or after osimertinib suggests that dual blockade of EGFR and MET by amivantamab can potentiate the initial anti-EGFR activity of lazertinib and may delay development of resistance through EGFR secondary resistance mutations and MET bypass pathways^41^, although direct comparison with single-agent lazertinib was not performed in this phase 1 trial. Additional studies to corroborate the results are currently underway. The CHRYSALIS-2 study (NCT04077463) is evaluating the amivantamab and lazertinib regimen in the post-platinum-based chemotherapy/post-osimertinib setting, with results demonstrating a consistent level of anti-tumor activity (ORR = 33% by blinded independent central review, with duration of response of 9.6 months)^42^, suggesting similar efficacy as observed in this current analysis. Similarly to this analysis, cohort D of the CHRYSALIS-2 study (NCT04077463) is investigating potential biomarker strategies and evaluating the amivantamab and lazertinib regimen in the post-osimertinib and chemotherapy-naive setting. In conclusion, the amivantamab and lazertinib regimen showed durable clinical activity in the osimertinib-relapsed setting, consistent with preclinical studies, suggesting improved anti-EGFR activity in osimertinib-resistant models. Exploratory NGS and IHC-based analyses suggest that these may represent biomarker strategies with the potential to enrich for a population of patients who are more likely to respond to the amivantamab and lazertinib regimen, and efforts to confirm these exploratory findings are ongoing.

## Methods

### Study design

CHRYSALIS is an ongoing, first-in-human, open-label, multicenter, dose-escalation (part 1) and dose-expansion (part 2) phase 1 study of amivantamab as monotherapy and as combination therapy in patients with advanced NSCLC (ClinicalTrials.gov identifier: NCT02609776). Details on the monotherapy study design were previously described^31^. For the amivantamab and lazertinib regimen (Fig. 1b), eligible patients had Eastern Cooperative Oncology Group performance status (ECOG PS) ≤1 and metastatic or unresectable NSCLC that was positive for *EGFR* ex19del or exon 21 L858R mutation based on local or central testing of ctDNA or tumor. For part 2, additional eligibility criteria included measurable disease according to Response Evaluation Criteria in Solid Tumors (RECIST) and disease that progressed after first-line or second-line treatment with a third-generation EGFR TKI (referred to as the osimertinib-relapsed cohort; previous progression on lazertinib was not exclusionary). Key exclusion criteria included previous treatment with anti-cancer immunotherapy for patients enrolled in the treatment-naive cohort and any previous treatment in the metastatic setting with therapy other than a first-generation, second-generation or third-generation EGFR TKI for the osimertinib-relapsed cohort (fewer than two cycles of platinum-based chemotherapy administered before the first EGFR TKI was allowed). Patients with untreated or asymptomatic brain metastases smaller than 1 cm in diameter at screening were eligible.

Part 1 dose escalation was implemented using a 3 + 3 design. Dosing was initiated at a dose level below the RP2D of amivantamab (700 mg for body weight <80 kg and 1,050 mg for body weight ≥80 kg) in combination with the RP2D of lazertinib (240 mg) and escalated to a second dose level of 1,050 mg of amivantamab for body weight <80 kg and 1,400 mg for body weight ≥80 kg in combination with 240 mg of lazertinib. Amivantamab was administered intravenously weekly during cycle 1 (28-d cycle) and then every other week thereafter. The first dose of amivantamab was split over 2 d, with 350 mg given on cycle 1, day 1 and the remainder of the full dose given on cycle 1, day 2. Lazertinib was given orally daily. The primary objective for part 1 was to determine the RP2CD. The primary objectives for part 2 were to evaluate the safety, tolerability and anti-tumor activity (ORR) of the amivantamab and lazertinib regimen at the RP2CD. Key secondary objectives included assessment of the clinical benefit, PFS and OS of the amivantamab and lazertinib regimen, and exploratory objectives included exploration of biomarkers predictive of clinical response from blood and tumor tissue.

Doses of amivantamab were administered intravenously once weekly for the first 4 weeks and then every other week for week 5 and beyond (Fig. 1b). To mitigate IRRs, the initial dose of amivantamab was given as a split dose of 350 mg on day 1 and the remainder of the dose on day 2. Lazertinib was given orally daily and before initiation of amivantamab infusion on days when amivantamab was also administered (Fig. 1b). Monitoring for IRRs during the initial dose and proactive infusion modifications were implemented to help mitigate IRRs^31,43^. Treatment continued until disease progression, unacceptable toxicity or withdrawal of consent. Treatment beyond RECIST-defined disease progression was allowed in cases of continued clinical benefit. Management of rash was recommended per protocol or in accordance with institutional guidelines^31^. The study was approved by institutional review boards at participating sites (Supplementary Table 8), and all patients provided written informed consent. The study was conducted in accordance with current International Council for Harmonization guidelines on Good Clinical Practice, consistent with the principles of the Declaration of Helsinki. Sex/gender was determined based on self-report.

### Study assessments

Disease was assessed by the investigator using computed tomography scans of the chest, abdomen, pelvis and any other disease location performed with intravenous contrast. Baseline brain magnetic resonance imaging was required at screening for patients enrolled in the dose-expansion cohort. Monitoring for CNS disease was performed in accordance with local practice. Tumor response was assessed by the investigator using RECIST version 1.1. AEs were graded according to National Cancer Institute Common Terminology Criteria for Adverse Events version 4.03.

### Statistical analysis

The data cutoff date for this analysis was 19 April 2021. The protocol-defined final analysis for the osimertinib-relapsed cohort was to occur after enrollment of 100 patients; however, guidance from health authorities limited enrollment to 45 patients for this first-in-human study, which was opened under a single-agent investigational new drug (IND). Under the direction of health authorities, CHRYSALIS-2 (NCT04077463) was opened under a combination IND, which allowed for the recruitment of a larger patient population. Therefore, the analysis presented here is a final exploratory analysis that includes the 45 patients who were enrolled in the osimertinib-relapsed cohort (also known as cohort E). The safety population included patients who were treated with the amivantamab and lazertinib regimen across both parts of the study (patients from all three cohorts; Fig. 1a). The efficacy population for each cohort included patients who were treated with the amivantamab and lazertinib regimen and had at least two scheduled post-baseline disease assessments or had discontinued treatment for any reason.

ORR was calculated as the proportion of patients in the efficacy population who achieved CR or PR as assessed by the investigator using RECIST version 1.1. The null hypothesis for cohort E was ORR ≤25%, and the alternative hypothesis was ORR ≥40%. A sample size of 100 response-evaluable patients, assuming a non-evaluable rate of 10%, was needed for a power of 85% and a one-sided alpha of 2.5%; however, because the study stopped enrollment prematurely, hypothesis testing was not performed. CBR was calculated as the proportion of patients achieving CR or PR or SD for ≥11 weeks, corresponding to two disease assessments.

Data were summarized using descriptive statistics. Observed ORR and CBR are presented along with their two-sided 95% CIs. The 95% CIs were calculated using log transformation, assuming the log (survival rate) is a normal distribution. Time to event endpoints were summarized using Kaplan–Meier estimates and presented with their corresponding 95% CIs.

### Biomarker analyses

NGS of pre-treatment tumor biopsies and plasma ctDNA were performed to elucidate the landscape of genomic alterations in patient tumors. Plasma samples were collected prospectively, before treatment, and were analyzed with Guardant360 (Guardant Health). Tumor biopsies were collected after progression on last anti-cancer therapy and before treatment with the amivantamab and lazertinib regimen. Tumor biopsy NGS was performed with the Oncomine Dx Target Test (Thermo Fisher Scientific).

Expression of EGFR and MET on available patient tumor samples was measured by IHC analysis of formalin-fixed, paraffin-embedded tumor tissue collected after progression on last anti-cancer therapy and before treatment with the amivantamab and lazertinib regimen. Staining for MET was performed with the anti-MET rabbit monoclonal antibody SP44; samples were run on the Dako Link 48 autostainer with FLEX detection. Staining for EGFR was performed with the anti-EGFR rabbit monoclonal antibody D38B1. Tumor cell staining was determined by the H-score method, as previously described^44^. IHC analysis was performed at Mosaic Laboratories. IHC-positive was defined as having a combined H-score >400 based on a response operator curve analysis revealing that the combined H-score of 400 was found to optimize both sensitivity and specificity for predicting response to amivantamab and lazertinib combination therapy. Individual H-scores for each receptor (EGFR and MET) were also evaluated; a score of 150 for each receptor indicated that it was probably driven by relatively high H-scores of both receptors rather than predominantly by a high H-score of one receptor but not the other. These H-score cutoffs were derived retrospectively and based on the approaches of previous studies^45–47^; prospective clinical validation of this cutoff is required and is currently underway.

### Reporting summary

Further information on research design is available in the Nature Portfolio Reporting Summary linked to this article.

## Online content

Any methods, additional references, Nature Portfolio reporting summaries, source data, extended data, supplementary information, acknowledgements, peer review information; details of author contributions and competing interests; and statements of data and code availability are available at 10.1038/s41591-023-02554-7.

### Supplementary information

Supplementary Information Supplementary Tables 1–8 and representative images of IHC staining panels **a** and **b.**

Reporting Summary

## Data Availability

Janssen has an agreement with the Yale Open Data Access (YODA) project to serve as the independent review panel for the evaluation of requests for clinical study reports and participant-level data from investigators and physicians for scientific research that will advance medical knowledge and public health. The project does not support requests to use data for non-scientific purposes, such as in pursuit of litigation or for commercial interests. Data will be made available after publication and approval by YODA of any formal requests with a defined analysis plan. For more information on this process or to make a request, visit the YODA project site at http://yoda.yale.edu (median response time for inquiries is 15 d). The data-sharing policy of Janssen Pharmaceutical Companies of Johnson & Johnson is available at https://www.janssen.com/clinical-trials/transparency.
