# Supplementary material for: Amivantamab plus lazertinib in osimertinib-relapsed EGFR-mutant advanced non-small cell lung cancer: a phase 1 trial
Source: Nat Med. 2023 Sep 14;29(10):2577–85. doi: 10.1038/s41591-023-02554-7 (PMC10579096; doi:10.1038/s41591-023-02554-7)
Supplement: Supplementary file 1 — Supplementary Tables 1–8 and representative images of IHC staining panels a and b. [file 41591_2023_2554_MOESM1_ESM.pdf]

nature medicine

Article

https://doi.org/10.1038/s41591-023-02554-7

Amivantamab plus lazertinib in osimertinib-relapsed *EGFR*-mutant advanced non-small cell lung cancer: a phase 1 trial

In the format provided by the authors and unedited

SUPPLEMENT

Table S1. Demographic and Baseline Disease Characteristics

	**Combination Cohort (n=26)**
Median age, yr (min–max)	60 (36 – 78)
Sex	
Female	16 (62)
Male	10 (38)
Race	
Asian	26 (100)
White	0
Black	0
Multiple / not reported	0
ECOG PS	
0	10 (38)
1	16 (62)
History of smoking	
Yes	12 (46)
No	14 (54)
Median time from initial diagnosis to first dose, months (min–max)	31 (1 – 75)
Location of metastases[a]	
Lymph node	9 (35)
Bone	3 (12)
Brain	9 (35)
Liver	1 (4)
Adrenal gland	0
Other / not reported	17 (65)
Median prior lines of therapy (min–max)	2 (0 – 9)

Prior systemic therapy	
Platinum-based chemotherapy[b]	11 (42)
EGFR TKI[a]	
1st or 2nd-generation	21 (81)
3rd-generation	8 (31)
No prior therapy	3 (12)

Data are number of patients (%) unless otherwise noted.

[a]Patients could be counted in more than one category.

[b]For the dose escalation cohort (n=26), there were no restrictions on prior therapies; the additional 7 patients in the all-treated population had less than 2 cycles of platinum-based chemotherapy (duration 22-65 days).

ECOG PS, Eastern Cooperative Oncology Group performance status; EGFR, epidermal growth factor receptor; NSCLC, non-small cell lung cancer; TKI, tyrosine kinase inhibitor.

Table S2. Adverse Events

Adverse events (≥10%), n (%)	Dose Escalation Cohort (n=26)	
	**All-grade**	**Grade ≥3**
Skin and subcutaneous tissue disorders		
Rash[a]	25 (96)	2 (8)
Pruritus	11 (42)	0
Dry skin	3 (12)	0
General disorders and administration site conditions		
Infusion-related reaction	16 (62)	1 (4)
Edema[b]	7 (27)	0
Fatigue[c]	6 (23)	0
Pyrexia	5 (19)	0
Infections and infestations		
Paronychia	19 (73)	2 (8)
Conjunctivitis	2 (8)	0
Metabolism and nutrition disorders		
Hypoalbuminemia	14 (54)	1 (4)
Decreased appetite	9 (35)	0
Hypocalcemia	4 (15)	1 (4)
Hypomagnesemia	3 (12)	0
Hyponatremia	3 (12)	3 (12)
Musculoskeletal and connective tissue disorders		
Musculoskeletal pain[d]	10 (38)	0
Gastrointestinal disorders		
Stomatitis[e]	11 (42)	0
Nausea	6 (23)	1 (4)
Constipation	7 (27)	0
Diarrhea	6 (23)	1 (4)
Dyspepsia	6 (23)	0
Abdominal pain	4 (15)	0

Anal inflammation	1 (4)	0
Hemorrhoids	0	0
Investigations		
Increased ALT	8 (31)	2 (8)
Increased AST	8 (31)	1 (4)
Increased GGT	2 (8)	1 (4)
Increased CPK	1 (4)	0
Nervous system disorders		
Paresthesia	12 (46)	0
Dizziness	7 (27)	0
Respiratory, thoracic, and mediastinal disorders		
Dyspnea[f]	1 (4)	1 (4)
Pleural effusion	4 (15)	1 (4)
Pulmonary embolism	3 (12)	0
Cough[g]	2 (8)	0
Vascular disorders		
Hemorrhage[h]	2 (8)	0
Blood and lymphatic system disorders		
Anemia	3 (12)	2 (8)
Renal and urinary disorders		
Acute kidney injury	0	0
Hepatobiliary disorders		
Cholecystitis	0	0

[a]Rash includes acne, dermatitis, dermatitis acneiform, eczema, eczema asteatotic, palmar-plantar erythrodysesthesia syndrome, perineal rash, rash, rash erythematous, rash maculo-papular, rash papular, rash vesicular, skin exfoliation, and toxic epidermal necrolysis

[b]Edema includes eyelid edema, face edema, generalized edema, lip edema, edema, edema peripheral, periorbital edema, peripheral swelling, oedema, oedema peripheral

[c]Fatigue includes asthenia and fatigue

[d]Musculoskeletal pain includes arthralgia, arthritis, back pain, bone pain, musculoskeletal chest pain, musculoskeletal discomfort, musculoskeletal pain, myalgia, neck pain, non-cardiac chest pain, pain in extremity, spinal pain

[e]Stomatitis includes aphthous ulcer, cheilitis, glossitis, mouth ulceration, mucosal inflammation, pharyngeal inflammation, stomatitis

[f]Dyspnea includes dypsnea and dyspnea exertional

[g]Cough includes cough, productive cough, and upper airway cough syndrome

[i]Hemorrhage includes epistaxis, gingival bleeding, hematuria, hemoptysis, hemorrhage, mouth hemorrhage, mucosal hemorrhage

ALT, alanine aminotransferase; AST, aspartate aminotransferase; CPK, creatine phosphokinase; GGT, gamma-glutamyltransferase

Table S3. Identified Alterations Among Patients with EGFR and/or MET-based Osimertinib Resistance

**Resistance**[a]	**Alterations**[b]
EGFR-based	C797S (n=7)<br>Amp (n=3)<br>L718X (n=3)<br>G724S (n=2)<br>L792H (n=1)<br>G796S (n=1)<br>E709K (n=1)
MET-based	Amp (n=5)<br>METex14 (n=1)
Additional	PIK3CA E542X (n=2)<br>CCNE1 Amp (n=1)<br>PIK3CA Amp (n=1)<br>CCND1 Amp (n=1)<br>CDK4 Amp (n=1)<br>KRAS Amp (n=1)<br>FGFR3-TACC3 fusion (n=1)<br>KRAS G12D (n=1)<br>CDKN2A G101W (n=1)

[a]EGFR amp (CNV ≥7) and MET amp (CNV ≥3) were based on tumor NGS; other amps were based on tumor NGS (CNV ≥7) or ctDNA NGS (CNV ≥3). Single nucleotide variants, insertion/deletions, and insertion call threshold was ≥1% allele frequency with >250 reads.

[b]Patients could be counted in more than one category; 8 patients had ≥1 alteration.

Amp, amplification; CCND1, cyclin D1; CCNE1, cyclin E1; CDK4, cyclin dependent kinase 4; CDKN2A, cyclin dependent kinase inhibitor 2A; EGFR, epidermal growth factor receptor; FGFR3-TACC3, fibroblast growth factor receptor 3–transforming acidic coiled coil-containing protein 3; KRAS, Kirsten rat sarcoma virus; PIK3CA, phosphatidylinositol-4,5-bisphosphate 3-kinase catalytic subunit alpha

Table S4 Best Response and Associated Biomarker Data of the Osimertinib-relapsed Cohort

Patient	Best Response[a]	EGFR/MET-based Resistance		EGFR/MET-independent Resistance		EGFR and MET IHC staining		
		Plasma	Tumor	Plasma	Tumor	IHC evaluable?	EGFR H score	MET H score
1	PD	-	n.d.	PIK3CA E545K	n.d.	n	-	-
2	NE/UNK	-	-	-	-	y	2	0
3	NE/UNK	-	-	-	-	y	70	110
4	NE/UNK	-	n.d.	-	n.d.	n	-	-
5	PD	-	EGFR Amp (CNV=14)	CCNE1 amp; PIKC3CA amp	-	y	36	3
6	PD	-	-	PTEN N48K; Alk-SQSTM1 fusion	-	n	-	-
7	PD	-	n.d.	KRAS A18V	n.d.	n	-	-
8	PD	-	n.d.	-	n.d.	n	-	-
9	SD	-	n.d.	CCND2 amp	n.d.	n	-	-
10	SD	-	-	-	CCND1 amp	y	210	0
11	SD	-	n.d.	-	n.d.	n	-	-
12	PD	EGFR C797S	EGFR C797S	-	-	n	-	-
13	PD	-	n.d.	PIK3CA E545K; KRAS G12C	n.d.	n	-	-
14	SD	-	n.d.	-	n.d.	n	-	-
15	NE/UNK	-	n.d.	PIK3CA H1047R	n.d.	n	-	-
16	PD	-	-	-	-	y	250	101
17	PD	-	-	PTEN I33del	-	n	-	-
18	PD	-	-	-	CCND1 amp	n	-	-
19	SD	EGFR C797S	n.d.	-	n.d.	n	-	-

20	SD	EGFR C797S	-	FGFR3-TACC3 fusion	-	n	-	-
21	SD	-	-	-	-	n	-	-
22	SD	-	n.d.	PIK3CA E545K	n.d.	n	-	-
23	SD	EGFR C797S	MET amp (CNV=4)	-	-	y	75	300
24	SD	EGFR C797S	-	KRAS G12D	-	y	60	5
25	SD	-	n.d.	-	n.d.	n	-	-
26	PD	MET Exon 14 skip	-	-	-	y	10	70
27	SD	EGFR C797S; EGFR L718Q	MET amp (CNV=7)	PIK3CA H1047L; PIK3CA H1047R	KRAS amp	n	-	-
28	SD	EGFR G724S	EGFR G724S	-	-	y	180	292
29	SD	-	-	-	-	y	102	19
30	PR	-	-	-	-	y	290	300
31	PR	EGFR C797S	EGFR C797S	-	-	n	-	-
32	PR	EGFR L718V; EGFR L718Q	EGFR L718Q	PIK3CA E542V	PIK3CA E542V	y	175	295
33	PR	n.d.	n.d.	n.d.	n.d.	n	-	-
34	PR	-	MET amp (CNV=31)	-	-	y	280	300
35	PR	-	n.d.	-	n.d.	n	-	-
36	PR	EGFR G796S	EGFR E709K; EGFR amp (CNV=37)	-	PIK3CA E542K; CCND1 amp; CDK4 amp	y	300	150
37	PR	-	-	-	-	y	250	185
38	PR	-	-	-	-	y	299	220
39	PR	-	-	-	-	y	270	300
40	PR	EGFR L792H	n.d.	-	n.d.	n	-	-
41	PR	-	n.d.	-	n.d.	n	-	-

42	PR	EGFR G724S	EGFR G724S;<br>EGFR amp (CNV=8)	CDKN2A G101W	-	n	-	-
43	PR	-	-	-	-	y	150	295
44	PR	-	MET amp (CNV=3)	-	-	y	1	175
45	CR	-	MET amp (CNV=3)	-	-	y	159	300

[a]All PR and CR were confirmed.

Amp, amplification; CCND1, cyclin D1; CCNE1, cyclin E1; CDK4, cyclin dependent kinase 4; CDKN2A, cyclin dependent kinase inhibitor 2A; EGFR, epidermal growth factor receptor; FGFR3-TACC3, fibroblast growth factor receptor 3–transforming acidic coiled coil-containing protein 3; KRAS, Kirsten rat sarcoma virus; n.d, not done; PIK3CA, phosphatidylinositol-4,5-bisphosphate 3-kinase catalytic subunit alpha

Table S5. Investigator-assessed Response by Biomarker Analysis in the Osimertinib-relapsed Cohort

	NGS-based Testing (n=45)		IHC-based Testing (n=20)	
	**EGFR/MET-based Resistance (n=17)**	**Non-EGFR/MET-based or Unknown Resistance (n=28)**	**IHC-positive (n=10)**	**IHC-negative (n=10)**
ORR[a] (95% CI)	47% (23 – 72)	29% (13 – 49)	90% (56 – 100)	10% (0.3 – 45)
CBR[b] (95% CI)	82% (57 – 96)	54% (34 – 73)	100% (69 – 100)	50% (19 – 81)
mDOR, months (95% CI)	10.4 (2.7 – NC)	8.3 (2.6 – NC)	9.7 (2.6 – NC)	2.7 (NC – NC)
mPFS, months (95% CI)	6.7 (3.4 – 12.5)	4.1 (1.4 – 9.5)	12.5 (4.0 – NC)	4.0 (1.4 – 4.4)

[a]Proportion of patients who had partial and complete responses.

[b]Proportion of patients who had partial and complete responses or stable disease for at least 11 weeks (corresponding to two disease assessments).

CBR, clinical benefit rate; CI, confidence interval; mDOR, median duration of response; mOS, median overall survival; mPFS, median progression-free survival; NC, not calculable; ORR, overall response rate; RECIST, Response Evaluation Criteria in Solid Tumors

Table S6. Best Response in Patients with Identified EGFR and/or MET-based Osimertinib Resistance Mechanisms

Patient	EGFR and/or MET-based Osimertinib Resistance Mechanism[a]	Additional Alterations[a]	EGFR H Score	MET H Score	Best Response
1	MET Amp (CNV=4)		159	300	CR
2	MET Amp (CNV=3)		1	175	PR
3	EGFR G724S, EGFR Amp (CNV=8)	CDKN2A G101W	Not performed	Not performed	PR
4	EGFR G796S, EGFR E709K, EGFR Amp (CNV=37)	CCND1 Amp, CDK4 Amp, PIK3CA E542K	300	150	PR
5	EGFR L792H		Not performed	Not performed	PR
6	MET Amp (CNV=31)		280	300	PR
7	EGFR L718Q, EGFR L718V	PIK3CA E542V	175	295	PR
8	EGFR C797S[b] (plasma + tissue)		Not performed	Not performed	PR
9	EGFR G724S		180	292	SD
10	EGFR C797S[b] (plasma only), EGFR L718Q (plasma only), MET Amp (CNV=7)	KRAS Amp	Not performed	Not performed	SD
11	EGFR C797S[b] (plasma only)	KRAS G12D	60	5	SD
12	EGFR C797S[b] (plasma only), MET Amp (CNV=4)		75	300	SD
13	EGFR C797S[b] (plasma only)	FGFR3-TACC3 Fusion	Not performed	Not performed	SD
14	EGFR C797S[b] (plasma only; tumor not tested)		Not performed	Not performed	SD
15	MET Exon 14		10	70	PD
16	EGFR C797S[b] (plasma + tumor)		Not performed	Not performed	PD
17	EGFR Amp (CNV=14)	CCNE1 Amp, PIK3CA Amp	36	3	PD

[a]EGFR amp (CNV ≥7) and MET amp (CNV ≥3) were based on tumor NGS; other amps were based on tumor NGS (CNV ≥7) or ctDNA NGS (CNV ≥3). Single nucleotide variants, insertion/deletions, and insertion call threshold was ≥1% allele frequency with >250 reads.

[b]All C797S mutations were of the cis configuration.

Amp, amplification; CCND1, cyclin D1; CCNE1, cyclin E1; CDK4, cyclin dependent kinase 4; CDKN2A, cyclin dependent kinase inhibitor 2A; EGFR, epidermal growth factor receptor; FGFR3-TACC3, fibroblast growth factor receptor 3–transforming acidic coiled coil-containing protein 3; KRAS, Kirsten rat sarcoma virus; PIK3CA, phosphatidylinositol-4,5-bisphosphate 3-kinase catalytic subunit alpha

Table S7. Identified Alterations Among Patients without EGFR and/or MET-based Osimertinib Resistance

**Resistance**	**Alterations**[a]
EGFR/MET-independent (n=10)	PIK3CA E545K (n=3)<br>CCND1 Amp (n=2)<br>CCND2 Amp (n=1)<br>KRAS A18V (n=1)<br>KRAS G12C (n=1)<br>PIK3CA H1047R (n=1)<br>PTEN I33del (n=1)<br>PTEN N48K (n=1)<br>SQSTM1-ALK fusion (n=1)
Not identified (n=18)	

[a]Patients could be counted in more than one category; 2 patients had ≥1 alteration

Amp, amplification; CCND1, cyclin D1; CCND2, cyclin D2; KRAS, Kirsten rat sarcoma virus; PIK3CA, phosphatidylinositol-4,5-bisphosphate 3-kinase catalytic subunit alpha; PTEN, phosphatase and tensin homolog deleted on chromosome 10; SQSTM1-ALK, sequestosome 1- anaplastic lymphoma kinase

Table S8. Participating Study Sites and Associated Institutional Review Boards

Participating Study Site	City	Institutional Review Board
Austin Hospital	Heidelberg, Australia	Austin Health Human Research Ethics Committee<br>----------------<br>Austin Health Office for Research
University Health Network	Toronto, Canada	UHN Research Ethics Board
Hosp. Univ. Quiron Dexeus	Barcelona, Spain	Comité Regional de la Comunidad de Madrid
Hosp. Gral. Univ. Gregorio Maranon	Madrid, Spain	Comité Regional de la Comunidad de Madrid
Royal Marsden Hospital	Sutton, UK	Bristol HRA Centre<br>----------------<br>North West - Haydock Research Ethics Committee<br>----------------<br>R&D Office, The Royal Marsden
Samsung Medical Center	Seoul, Republic of Korea	Samsung Medical Center Institutional Review Board
Seoul National University Hospital	Seoul, Republic of Korea	Seoul National University Hospital Institutional Review Board
Severance Hospital, Yonsei University Health System	Seoul, Republic of Korea	Severance Hospital Institutional Review Board
Gachon University Gil Medical Center	Incheon, Republic of Korea	Gachon University Gil Medical Center Institutional Review Board
Chungbuk National University Hospital	Cheongju-si, Republic of Korea	Chungbuk National University Hosptial Institutional Review Board
Asan Medical Center	Seoul, Republic of Korea	Asan Medical Center
H. Lee Moffitt Cancer & Research Institute	Tampa, FL, USA	Chesapeake Institutional Review Board<br>----------------<br>Advarra
City of Hope	Duarte, CA, USA	Western Institutional Review Board

University of Pennsylvania Division of Hematology Oncology Perelman Center for Advanced Medicine	Philadelphia, PA, USA	University of Pennsylvania Office of Regulatory Affairs Institutional Review Board
Dana Farber Cancer Institute	Boston, MA, USA	Dana-Farber Cancer Institute Institutional Review Board
Samuel Oschin Comprehensive Cancer Center Cedars-Sinai Medical Center	West Hollywood, CA, USA	Cedars Sinai Office of Research Compliance and Quality Improvement
Langone Health at NYC University, NYU School of Medicine	New York, NY, USA	NYU School of Medicine Institutional Review Board
Providence Portland Medical Center	Portland, OR, USA	Providence St. Joseph Health Institutional Review Board
Virginia Cancer Specialists	Fairfax, VA, USA	Advarra
Icahn School of Medicine at Mt. Sinai	New York, NY, USA	Icahn School of Medicine at Mount Sinai-Program for the Protection of Human Subjects
UCLA	Santa Monica, CA. USA	UCLA Office of Human Research Protection Program
Barbara Ann Karmanos Cancer Institute	Detroit, MI, USA	Western Institutional Review Board
University of Chicago	Chicago, IL, USA	University of Chicago Institutional Review Board
Chao Family Comprehensive Cancer Center	Orange, CA, USA	UC Irvine Institutional Review Board
Oncology Consultants - Texas	Houston, TX, USA	Advarra

Representative Images of IHC Staining

A. IHC Positive

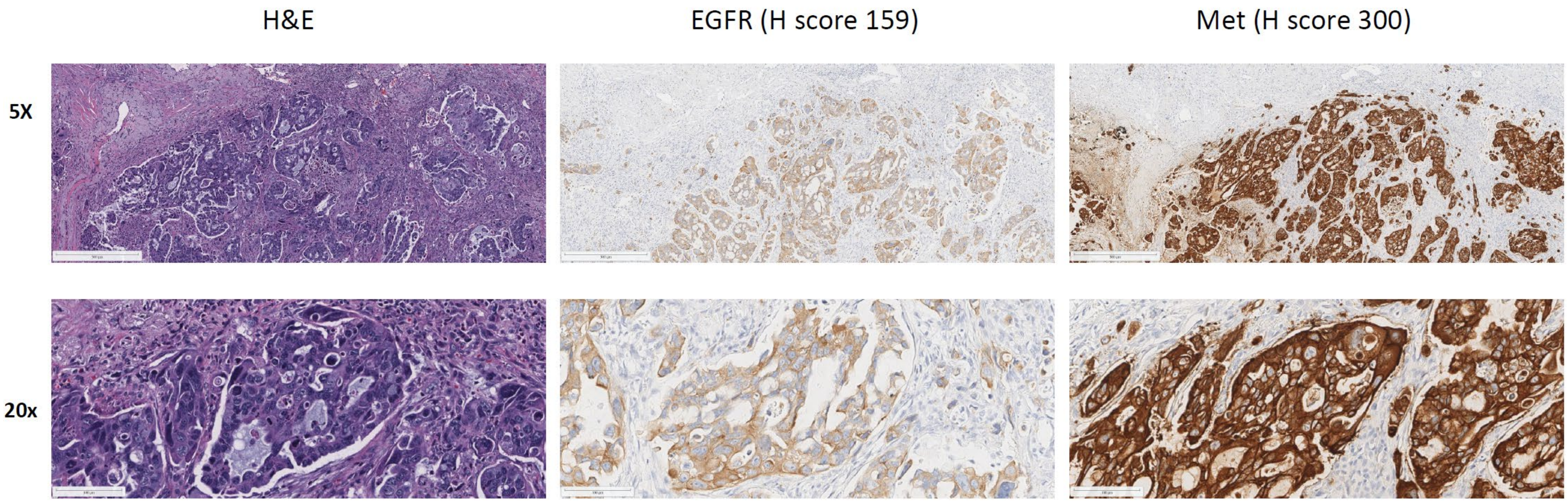

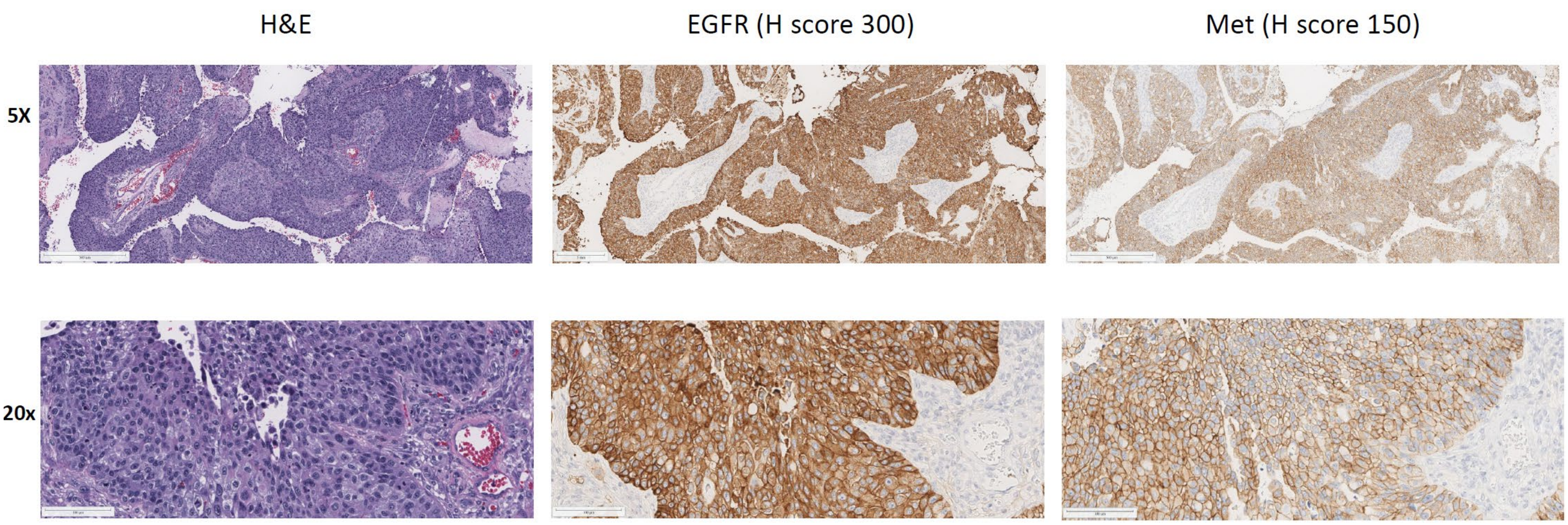
H&E
EGFR (H score 300)
Met (H score 150)
5X
20x

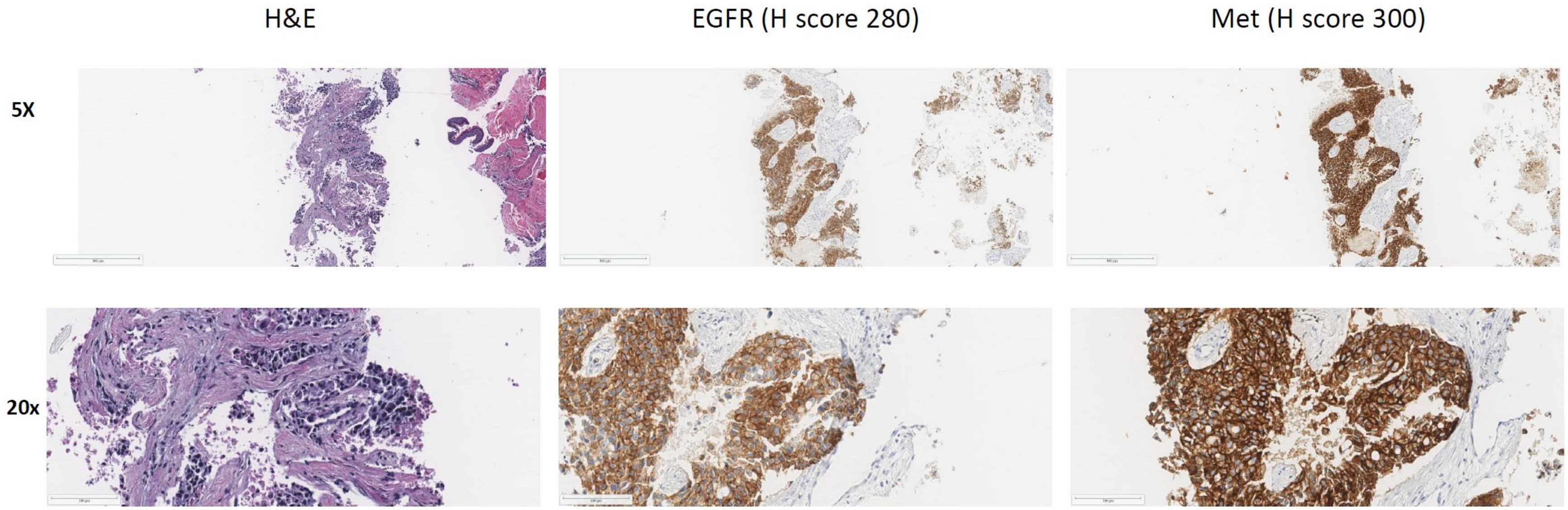
H&E
EGFR (H score 280)
Met (H score 300)
5X
20x

B. IHC Negative

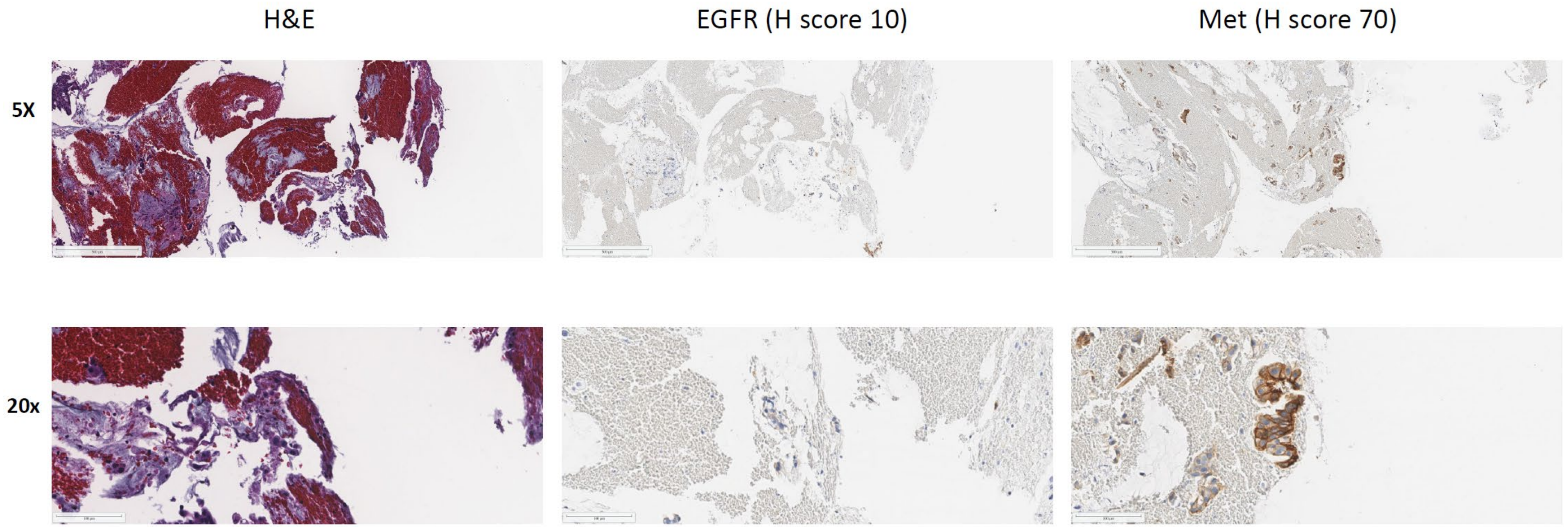

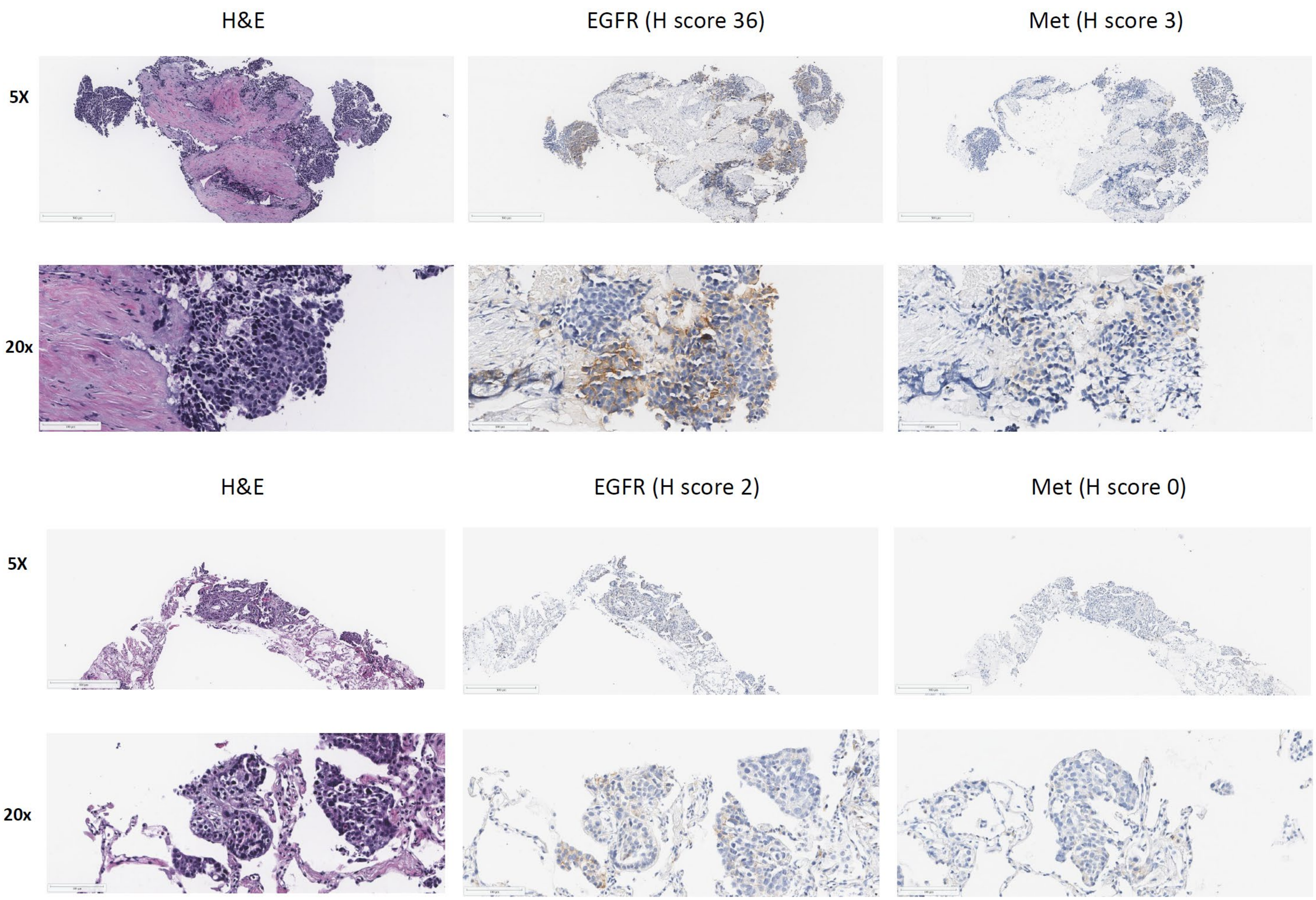
H&E
EGFR (H score 36)
Met (H score 3)
5X
20x
H&E
EGFR (H score 2)
Met (H score 0)
5X
20x
