# Supplementary material for: Amivantamab plus lazertinib in osimertinib-relapsed EGFR-mutant advanced non-small cell lung cancer: a phase 1 trial
Source: Nat Med. 2023 Sep 14;29(10):2577–85. doi: 10.1038/s41591-023-02554-7 (PMC10579096; doi:10.1038/s41591-023-02554-7)
Supplement: Supplementary file 2 — Reporting Summary [file 41591_2023_2554_MOESM2_ESM.pdf]

nature portfolio

Corresponding author(s): Byoung Chul Cho

Last updated by author(s): Aug 17, 2023

Reporting Summary

Nature Portfolio wishes to improve the reproducibility of the work that we publish. This form provides structure for consistency and transparency in reporting. For further information on Nature Portfolio policies, see our Editorial Policies and the Editorial Policy Checklist.

Statistics

For all statistical analyses, confirm that the following items are present in the figure legend, table legend, main text, or Methods section.

n/a	Confirmed	
☐	☒	The exact sample size (*n*) for each experimental group/condition, given as a discrete number and unit of measurement
☒	☐	A statement on whether measurements were taken from distinct samples or whether the same sample was measured repeatedly
☐	☒	The statistical test(s) used AND whether they are one- or two-sided<br>*Only common tests should be described solely by name; describe more complex techniques in the Methods section.*
☒	☐	A description of all covariates tested
☒	☐	A description of any assumptions or corrections, such as tests of normality and adjustment for multiple comparisons
☐	☒	A full description of the statistical parameters including central tendency (e.g. means) or other basic estimates (e.g. regression coefficient) AND variation (e.g. standard deviation) or associated estimates of uncertainty (e.g. confidence intervals)
☒	☐	For null hypothesis testing, the test statistic (e.g. *F*, *t*, *r*) with confidence intervals, effect sizes, degrees of freedom and *P* value noted<br>*Give P values as exact values whenever suitable.*
☒	☐	For Bayesian analysis, information on the choice of priors and Markov chain Monte Carlo settings
☒	☐	For hierarchical and complex designs, identification of the appropriate level for tests and full reporting of outcomes
☒	☐	Estimates of effect sizes (e.g. Cohen's *d*, Pearson's *r*), indicating how they were calculated

Our web collection on statistics for biologists contains articles on many of the points above.

Software and code

Policy information about availability of computer code

Data collection	No software was used.
Data analysis	No software was used.

For manuscripts utilizing custom algorithms or software that are central to the research but not yet described in published literature, software must be made available to editors and reviewers. We strongly encourage code deposition in a community repository (e.g. GitHub). See the Nature Portfolio guidelines for submitting code & software for further information.

Data

Policy information about availability of data

All manuscripts must include a data availability statement. This statement should provide the following information, where applicable:

- Accession codes, unique identifiers, or web links for publicly available datasets
- A description of any restrictions on data availability
- For clinical datasets or third party data, please ensure that the statement adheres to our policy

Janssen has an agreement with the Yale Open Data Access (YODA) Project to serve as the independent review panel for the evaluation of requests for clinical study reports and participant-level data from investigators and physicians for scientific research that will advance medical knowledge and public health. The project does not support requests to use data for non-scientific purposes, such as in pursuit of litigation or for commercial interests. Data will be made available following publication and approval by YODA of any formal requests with a defined analysis plan. For more information on this process or to make a request, please visit the

Yoda Project site at http://yoda.yale.edu (median response time for inquiries is 15 days). The data sharing policy of Janssen Pharmaceutical Companies of Johnson & Johnson is available at https://www.janssen.com/clinical-trials/transparency.

Human research participants

Policy information about studies involving human research participants and Sex and Gender in Research.

Reporting on sex and gender	Sex and/or gender was determined based on self-report. Demographic table reports breakdown by sex (female and male) of the patients. No sex- and gender-based analyses was performed because there was no priori biological rationale to indicate that sex or gender would have an impact on efficacy. Additionally, a subgroup analysis of efficacy and/or safety by sex/gender would be limited by sample size and could lead to misleading conclusions as a result.
Population characteristics	This global study enrolled patients with metastatic or unresectable NSCLC that was positive for EGFR ex19del or exon 21 L858R mutation based on local or central testing of ctDNA or tumor. Median age (range) was 65 (39-85) y for the osimertinib-relapsed patients and 61 (36-85) y for the all-treated population. Demographics are detailed in the manuscript text and Table 1. Subgroup analyses by covariates would be limited by sample size and could lead to misleading conclusions as a result.
Recruitment	This study was recruited globally, conducted at 25 number of sites. There may be a regional bias as recruited patients tended to occur more in South Korea (16 out of 45 patients enrolled). This study was started in South Korea, and the dose escalation was exclusively conducted in Korean subjects, while the combination cohort amendment was being expanded globally. In the expansion phase, the US was the primary recruiter. Although more patients were enrolled from South Korea, prior analyses of Asian subgroups with EGFR mutated NSCLC treated with amivantamab have shown comparable efficacy and safety.
Ethics oversight	The study was approved by participating site Institutional Review Boards (listed below and in the supplement) and all patients provided written informed consent.<br>1. Austin Hospital, Heidelberg, Australia; Austin Health Human Research Ethics Committee, Austin Health Office for Research<br>2. University Health Network, Toronto, Canada; UHN Research Ethics Board<br>3. Hosp. Univ. Quiron Dexeus, Barcelona, Spain; Comité Regional de la Comunidad de Madrid<br>4. Hosp. Gral. Univ. Gregorio Maranon, Madrid, Spain; Comité Regional de la Comunidad de Madrid<br>5. Royal Marsden Hospital, Sutton, UK; Bristol HRA Centre, North West; Haydock Research Ethics Committee; R&D Office, The Royal Marsden<br>6. Samsung Medical Center, Seoul, Republic of Korea; Samsung Medical Center Institutional Review Board<br>7. Seoul National University Hospital, Seoul, Republic of Korea; Seoul National University Hospital Institutional Review Board<br>8. Severance Hospital, Yonsei University Health System, Seoul, Republic of Korea; Severance Hospital Institutional Review Board<br>9. Gachon University Gil Medical Center, Incheon, Republic of Korea; Gachon University Gil Medical Center Institutional Review Board<br>10. Chungbuk National University Hospital, Cheongju-si, Republic of Korea; Chungbuk National University Hosptial Institutional Review Board<br>11. Asan Medical Center, Seoul, Republic of Korea; Asan Medical Center<br>12. H. Lee Moffitt Cancer & Research Institute, Tampa, FL, USA; Chesapeake Institutional Review Board, Advarra<br>13. City of Hope, Duarte, CA, USA; Western Institutional Review Board<br>14. University of Pennsylvania, Division of Hematology Oncology, Perelman Center for Advanced Medicine, Philadelphia, PA, Institutional Review Board<br>15. Dana Farber Cancer Institute, Boston, MA, USA; Dana-Farber Cancer Institute Institutional Review Board<br>16. Samuel Oschin Comprehensive Cancer Center Cedars-Sinai Medical Center, West Hollywood, CA, USA; Cedars Sinai Office of Research Compliance and Quality Improvement<br>17. Langone Health at NYC University, NYU School of Medicine, New York, NY, USA; NYU School of Medicine Institutional Review Board<br>18. Providence Portland Medical Center, Portland, OR, USA; Providence St. Joseph Health Institutional Review Board<br>19. Virginia Cancer Specialists, Fairfax, VA, USA; Advarra<br>20. Icahn School of Medicine at Mt. Sinai New York, NY, USA; Icahn School of Medicine at Mount Sinai-Program for the Protection of Human Subjects<br>21. UCLA, Santa Monica, CA. USA; UCLA Office of Human Research Protection Program<br>22. Barbara Ann Karmanos Cancer Institute, Detroit, MI, USA; Western Institutional Review Board<br>23. University of Chicago, Chicago, IL, USA; University of Chicago Institutional Review Board<br>24. Chao Family Comprehensive Cancer Center, Orange, CA, USA; UC Irvine Institutional Review Board<br>25. Oncology Consultants - Texas, Houston, TX, USA; Advarra

Note that full information on the approval of the study protocol must also be provided in the manuscript.

Field-specific reporting

Please select the one below that is the best fit for your research. If you are not sure, read the appropriate sections before making your selection.

☒ Life sciences ☐ Behavioural & social sciences ☐ Ecological, evolutionary & environmental sciences

For a reference copy of the document with all sections, see nature.com/documents/nr-reporting-summary-flat.pdf

Life sciences study design

All studies must disclose on these points even when the disclosure is negative.

Sample size	The null hypothesis is the ORR ≤25%, and the alternative hypothesis is the ORR ≥40%. With a one-sided alpha of 2.5%, and a power of 85%, the total sample size needed for the cohort is 93 response-evaluable subjects. Assuming a non-evaluable rate of 10%, a total of up to 100 subjects will be enrolled in the cohort. Guidance from health authorities limited enrollment to 45 patients for this first-in-human study.
Data exclusions	Inclusion/exclusion criteria are provided in the methods section. The goal of the study was assess preliminary efficacy in chemotherapy-naïve patients with EGFR ex19del or L858R NSCLC whose disease progressed on osimertinib or another third-generation EGFR TKI.
Replication	This was a clinical study, replication at present not available
Randomization	This was not a randomized study; this is a single-arm trial with no control arm.
Blinding	The study was unblinded; this is a single-arm trial with no control arm.

Reporting for specific materials, systems and methods

We require information from authors about some types of materials, experimental systems and methods used in many studies. Here, indicate whether each material, system or method listed is relevant to your study. If you are not sure if a list item applies to your research, read the appropriate section before selecting a response.

Materials & experimental systems

n/a	Involved in the study	
☐	☒	Antibodies
☒	☐	Eukaryotic cell lines
☒	☐	Palaeontology and archaeology
☒	☐	Animals and other organisms
☐	☒	Clinical data
☒	☐	Dual use research of concern

Methods

n/a	Involved in the study	
☒	☐	ChIP-seq
☒	☐	Flow cytometry
☒	☐	MRI-based neuroimaging

Antibodies

Antibodies used

Antibody 1: anti-EGFR
Supplier name: Cell Signaling
Catalog number: 4267
Clone name: D38B1
Lot number: 19 and 24

Antibody 1: anti-Met
Supplier name: Abcam
Catalog number: ab227637
Clone name: SP44
Lot number: GR3213935 and GR3213935-6

Validation

Antibody 1: anti-EGFR
Validation: anti-EGFR IHC validation efforts were undertaken using FFPE from human tissue. The IHC was validated to optimize signal-to-noise, assay sensitivity, specificity, precision and robustness using tonsil specimens, multiple cell lines with known EGFR expression levels, normal and tumor cores, and whole tissue sections and tumor sections. Results were confirmed through orthogonal IHC assay validation with a separate, commercially EGFR antibody.

Antibody 1: anti-Met
Validation: anti-Met IHC validation efforts were undertaken using FFPE from human tissue. The IHC was validated to optimize signal-to-noise, assay sensitivity, specificity, precision and robustness using control NSCLC specimens, multiple cell lines with known Met expression levels, normal tissue sections and tumor sections. Additionally, utilization of this IHC clone for detection of Met expression is reported widely across the literature (references already provided).

The IHC antibodies have been used by independent investigators across independent studies (EGFR D38B1: Niederst et al., Nat Comm 2015;6:6377; Simonetti et al., J Transl Med 2010;8:135; Kappler et al., Mol Clin Oncol 202;13(6)88. Met SP44: Guo et al., J Thorac Oncol. 2019;14(9):1666-1671; Boyle et al., Appl Immunohistochem Mol Morphol. 2020;28(9):669-677; Camidge et al., JTO Clin Res Rep 2021;3(1):100262; Stickler et al., J Clin Oncol. 2018;36(33):3298-3306). Furthermore, both assays passed Janssen and Vendor SME pathologist standards for assay validation.

Clinical data

Policy information about clinical studies

All manuscripts should comply with the ICMJE guidelines for publication of clinical research and a completed CONSORT checklist must be included with all submissions.

Clinical trial registration	Clinicaltrials.gov identifier: NCT02609776
Study protocol	Redacted protocol and statistical analysis plan provided in the supplement.
Data collection	Cohort E enrolled from December 3, 2019 until April 30, 2020, with a data cut as of April 19, 2021; study is currently ongoing. Study sites are listed in the supplement.
Outcomes	The protocol-specified primary objectives for the dose escalation phase were to evaluate the safety, tolerability, and antitumor activity (ORR) of the amivantamab and lazertinib regimen at the RP2CD. Protocol-specified key secondary objectives included assessment of the clinical benefit, PFS, and OS of the amivantamab and lazertinib regimen. AEs were graded according to National Cancer Institute Common Terminology Criteria for Adverse Events, version 4.03. Disease was assessed by the investigator using computed tomography scans of the chest, abdomen, pelvis, and any other disease location performed with IV contrast. Baseline brain magnetic resonance imaging was required at screening for patients enrolled in the dose expansion cohort. Monitoring for central nervous system disease was performed in accordance with local practice. Tumor response was assessed by the investigator using RECIST v1.1; overall response rate was the primary endpoint.
